# Investigation of Micro-volume Viscosity with Janus Microbeads Based on Rotational Brownian Motion

**DOI:** 10.3390/s19051217

**Published:** 2019-03-10

**Authors:** Chun-Jui Chen, Wei-Long Chen, Pham Hong Phong, Han-Sheng Chuang

**Affiliations:** 1 Department of Biomedical Engineering, National Cheng Kung University, Tainan 701, Taiwan; s8391016@gmail.com (C.-J.C.); alex821214@gmail.com (W.-L.C.); 2 Institute of Chemistry, Vietnam Academy of Science and Technology, Hanoi 1000, Vietnam; phphong@ich.vast.vn; 3 Graduate University of Science and Technology, Vietnam Academy of Science and Technology, Hanoi 1000, Vietnam; 4 Medical Device Innovation Center, National Cheng Kung University, Tainan 701, Taiwan

**Keywords:** rotational Brownian motion, Janus microbead, micro-volume viscosity, cross-correlation algorithm

## Abstract

Viscosity is an important property of liquids. A viscosity change of aqueous substances that deviates from their normal levels usually implies a compromise in quality due to degradation or microorganism proliferation. Monitoring of macro-scale viscosity can be simply realized by various conventional tools, such as rotational viscometers, capillary tubes, falling bodies, and so forth. Nevertheless, today, micro-volume viscosity measurement remains a challenging endeavor, resulting in rare, expensive, or difficult-to-obtain samples not very well studied. For this reason, a novel technique for micro-viscosity based on rotational Brownian motion is presented in this paper. Janus microbeads were made by coating fluorescent polystyrene beads with gold film. Taking advantage of the bead configuration of half gold/half fluorescence, the rotational Brownian signal was expressed in terms of blinking fluorescent intensity. The characteristic correlation time was derived from the blinking intensity of trace amounts of a selected medium over a certain time period, and results were correlated with viscosity. Given a volume of only 2 μL for each measurement, calibration of a series of glycerol–water mixtures (100%–1% (*v*/*v*) water content) yielded good agreement with the expected viscosity predictions over the range of 0.8–574.8 cP. Five common oil products, including lubricant oil, baby oil, food oil, olive oil, and motor oil, were further investigated to demonstrate the feasibility and practicability of the proposed technique. Data measured by the rotational Brownian motion-based diffusometer were comparable with those measured by a commercial rotational viscometer. The method also explicitly showed viscosity degradation after the oils were heated at a high temperature of over 100 °C for 10 min. Evaluation proved the proposed Janus microbead-enabled rotational diffusometric technique to be a promising approach for rapid and micro-scale viscosity measurement.

## 1. Introduction

Viscosity is a metric commonly used to evaluate the quality of liquids. A viscosity shift in a liquid medium can alter mixing efficiency, chemical reactions [1], and even the growth of microorganisms [2]. Accordingly, monitoring of viscosity is an essential step to ensure good quality control. In the food industry, the chemical structures of cooking oil change after repeated heating (e.g., deep frying) likely due to increases in saturated fatty acids [3,4]. Prior studies [5,6] have also indicated that oil degradation is noxious to human health due to massive formation of free radicals. In biology, the viscosity of physiological fluids varies in different samples. For example, blood and tissues can alter their physiological viscosity in response to environmental stimuli. As a result, the baseline viscosity is treated as an important sign of normality [7,8]. 

Micro-volume viscosity measurements in bulk or intercellular liquids are crucial when dealing with costly, rare, or difficult-to-obtain samples, such as a special paste for cerebral endovascular treatments [9], mouse blood [10], and fluids in insects [11]. Conventional viscometers, including rotational viscometers [12], capillary tubes [13], falling bodies [14], oscillating methods [15], and ultrasonic methods [16], have been deployed in many fields, but these devices are usually incapable of micro-volume (<several tens of microliters) viscosity measurement. To address the technical obstacles, numerous innovative techniques have been developed over the past decade [17,18]. Zhang et al. [19], for example, reported a method of local viscosity measurement (<30 μL) by evaluating the critical escape velocity of a poly(methyl methacrylate) (PMMA) bead trapped by an optical tweezer and found that the escape velocity has an inverse relationship with the liquid viscosity. However, the dynamic range of measurement of this technique was limited to low-viscosity liquids (<1.6 mPa·s) due to weak trapping forces. In addition, operations of the optical tweezer relied on highly trained personnel. Similar work with an integrated optofluidic chip was also conducted by Yang et al. [20]. Instead of trapping microbeads, they laterally shot single microbeads in a microchannel with optical fibers. In their study, fluid viscosities ranging from 1 mPa·s to 100 mPa·s and small sample volumes (<1 μL) were achieved. Kim et al. [21] proposed a micro-viscometer on a microchip with 10 array microchannels to achieve a wide-ranging shear rate; this device was used to estimate the dynamic viscosity of blood in different conditions, e.g., hematocrit. However, the microchip required flowing fluids and, thus, a large sample volume (at least several tens of microliters). Alternatively, Zhao et al. [22] fabricated a MEMS-based cantilever resonant microsensor to measure viscosity by immersing the whole device in a sample fluid. The resonant frequency showed a tendency to decline with increasing viscosity. However, the measurement region of interest was limited by the size of the cantilever beam (~hundreds of microliters), and the moving part could disrupt the local viscosity by imparting the contact liquid with extra energy. Instead of developing contact measurement, a continuous and non-intrusive approach was proposed [23] to monitor the time-varying viscosity of bacterial biofilms by simply combining video tracking with a semi-empirical flow model. Despite no external probes needed for targets, this approach lacked accuracy and could not be used in transparent media. In addition to intercellular liquids, rising endeavors have also been focused on the intracellular world due to the importance of single cell analysis. Chen et al. [24] recently investigated the cellular microviscosity of *Escherichia coli* by time-resolved linear dichroism spectroscopy. The cytoplasmic volume in cells is roughly less than 1 pL. Myoglobins (cMbCO) in the *E. coli* cells were excited by a linearly rotated light, and the subsequent rotational diffusion time was detected. The final microviscosity of the cytoplasm was found to be roughly threefold higher than that of water. Despite the promising applications of the device in live cells, however, it was excessively sophisticated and relied on some special proteins. Similar techniques focusing on the microviscosity of cells can be found in the literature [25,26,27,28,29]. Although intracellular viscosity measurement is not in the scope of our current study, researchers [29] have successfully demonstrated the potential of Brownian motion based on particle tracking for cellular microviscosity. By injecting nanobeads into the cytoplasm of HeLa cells, they achieved the study of intracellular viscoelasticity during cell division. According to their inspiring attempt, a similar study based on our platform can be realized in the same fashion by reducing the current Janus microbeads into nano-sized beads in the future.

Our previous research proved that translational Brownian motion may be a promising method in micro-volume viscosity measurement [30] and other applications [31,32,33,34,35]. Considering that Brownian motion is simply driven by thermal effects, it is independent of external power. The diffusivity of translational Brownian motion is inversely proportional to the suspended bead diameter according to the Stokes–Einstein relation [36]. By contrast, the diffusional signal can be further improved when rotational Brownian motion is dominant. According to the Stokes–Einstein–Debye relation [37], the diffusivity of rotational Brownian motion is inversely proportional to the cubic bead diameter. Therefore, the signal-to-noise ratio (SNR) can be further enhanced by rotational Brownian motion [38]. Unlike microrheometry [39] that can measure the viscoelasticity of materials, our diffusometer dealt with only viscosity due to simple targets (Newtonian fluids) investigated in the study. Nevertheless, the diffusometer can be extended to cope with viscosity and elasticity by performing frequency-related shear forces to liquids [29]. In the present study, Janus microbeads were fabricated by coating a thin layer of gold film on the surface of fluorescent polystyrene (PS) microbeads. After resuspending the Janus microbeads in a test medium, the cross-correlation algorithm was used to analyze consecutive changes in particle images over time. A characteristic correlation time was then derived from the exponential curve fitting of the time-elapsed correlation intensity. The correlation time increased with viscosity due to slow translational and rotational Brownian motion. A calibration curve was obtained by measuring glycerol–water mixtures with 1%–100% (*v*/*v*) water content. As a proof of concept, five frequently used oil products, including lubricant oil, baby oil, food oil, olive oil, and motor oil, were investigated with the proposed diffusometer, and results were compared with those measured by a rotational viscometer. Good agreement in trend between our diffusometer and the commercial viscometer was obtained. Another test was conducted by heating food oil and olive oil at a high temperature for 10 min to intentionally downgrade their quality. The viscosities of both oils tended to increase after heat treatment, thereby implying degradation. Between the oils, olive oil showed a lesser characteristic correlation time shift, indicating better durability against heat processing. Finally, the rotational diffusometer with Janus microbeads achieved measurement from 0.8 cP to 574.8 cP with a volume of only 2 μL per run. The limit of detection was as low as 0.8 cP. The proposed rotational diffusometer provides a generally promising alternative to current devices for micro-volume viscosity measurement.

## 2. Materials and Methods

### 2.1. Rotational Brownian Motion and Cross-Correlation Algorithm

**Figure 1:**
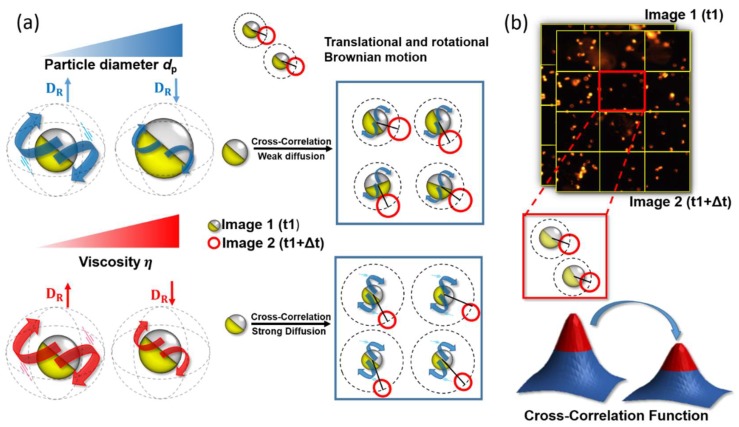
(**a**) Illustration of rotational Brownian motion in response to viscosity and bead size changes. Both bead size (top row) and viscosity (bottom row) result in translational and rotational Brownian motion changes. As demonstrated, the increase in the bead size or in the viscosity reduces Brownian motion of microbeads and vice versa. (**b**) Schematic of the cross-correlation algorithm. In the algorithm, each image is divided into multiple interrogation windows to facilitate computation. A pair of particle images (captured at different time frames) can yield a correlation function to express the degree of instantaneous Brownian motion.

Brownian motion can be classified into translational and rotational diffusion. The former is expressed in terms of random paths in space, while the latter is expressed in terms of random rotation spinning about an arbitrary axis (Figure 1a). According to the Stokes–Einstein relation [36], translational diffusivity can be written as:

(1) $$D_{t}=\frac{K_{B}T}{3\pi \mu d_{p}},$$

where *k_B_* is the Boltzmann constant, *T* is the absolute temperature, *μ* is the liquid viscosity, and *d_p_* represents the bead diameter. The rotational diffusivity defined by the Stokes–Einstein–Debye relation [37] is written as:

(2) $$D_{r}=\frac{K_{B}T}{\pi \mu d_{p}^{3}}.$$

Unlike translational Brownian motion, few studies have been carried out using rotational Brownian motion because extra effort is needed to detect the rotational diffusivity. Comparing Equations (1) and (2), rotational diffusivity is apparently more sensitive to viscosity changes than translational diffusivity as the bead size is reduced. To realize the measurement of rotational Brownian motion, Janus microbeads were used to generate the desired blinking signals. This blinking effect results from the jittering motion of microbeads suspended in a medium. High viscosity produces high friction acting on the surfaces of the microbeads, thereby lowering their angular velocity. Instead of tracking individual microbeads, in this work, we sequentially applied the cross-correlation algorithm on two particle images separated by a time interval *Δt* starting from 0 s to 50 s. The correlation intensity tends to decline as *Δt* increases (Figure 1b) due to the low degree of correlation between two particle images. The intensity was normalized by dividing the following intensity with the previous one. The time-elapsed correlation intensity was eventually approximated by exponential curve fitting as follows [40]:

(3) $$A\exp\left(-\frac{t}{\emptyset }\right)+B,$$

where *A* and *B* are constants determined by fitting of the exponential curve to the data, *t* is the elapsed time, and ø is the characteristic correlation time of this curve. The correlation time can be expressed as [40]:

(4) $$\emptyset =\frac{\mu V}{k_{B}T},$$

where *V* represents the equivalent volume of the microbeads. The correlation time is, therefore, proportional to the viscosity when the equivalent volume of the microbeads is fixed.

### 2.2. Measurement Concept and Experimental Setup 

**Figure 2:**
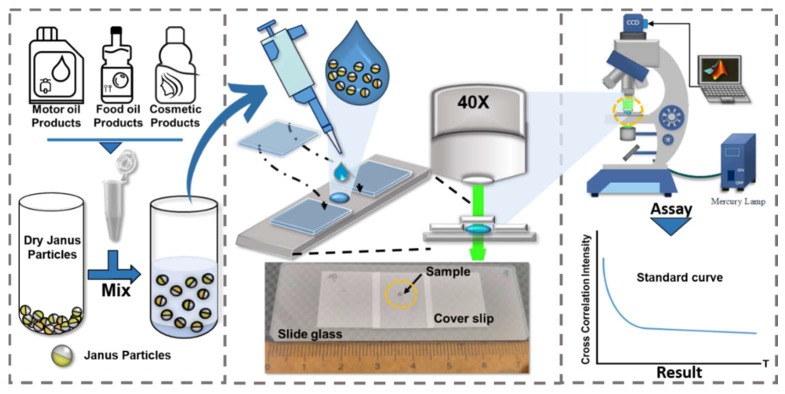
Conceptual illustration of the proposed rotational diffusometer and experimental setup. The rotational diffusometer is implemented with bead-based sensors made up of Janus microbeads (**left**). Only a small drop (2 μL) of Janus microbead suspension is required to be sandwiched between two glass slides before each measurement. A real image showing the microchip alongside a ruler displays its actual dimensions (**middle**). A fluorescent microscope is used to visualize the blinking Janus microbeads and acquire a series of particle images for computation of rotational Brownian motion (**right**).

According to the Stokes–Einstein–Debye relation, the Brownian motion of microbeads can directly reflect the viscosity of medium when the ambient temperature is well controlled. To this end, 1-μm Janus microbeads were thoroughly mixed with a test liquid medium in advance. Surfactant (5%, *v*/*v*) was added to the medium with oil, after which the colloidal suspension was sonicated for 1 min to ensure thorough dispersion of the microbeads. Exactly 2 μL of the suspension was pipetted onto a glass slide. A glass cover was then placed on top of the suspension droplet with a spacer of 170 μm. A thin thermal couple was also inserted into the same droplet to monitor its temperature. By positioning the sandwiched suspension droplet under a fluorescent microscope (IX 71, Olympus, Tokyo, Japan) with a 64× objective, a series of consecutive particle images were captured by a digital camera (Firefly MV FMVU-13S2C, Point Grey, Richmond, BC, Canada) at a frame rate of 23 Hz for 50 s (Figure 2). Notably, the frame rate was switched to 5 Hz to overcome a slow decline when viscosity was higher than 300 cP. Blinking signals measured from the Janus microbeads provided visual information of rotational Brownian motion.

Glycerol–water mixtures were prepared with different water contents ranging from 1% to 100% (*v*/*v*). The characteristic correlation times and predicted viscosities were plotted as a calibration curve. The final viscosities of other unknown samples were derived from the calibration curve. Subsequently, five oil products, including lubricant oil (DB, Gulf, London, UK), baby oil (Johnson’s, New Brunswick, NJ, USA), food oil (a combination of soybean oil, sunflower oil and rice oil; Neptune Gold, CALOFIC, Ho Chi Minh, Vietnam), olive oil (García de la Cruz, Toledo, Spain), and motor oil (Active 20W-40, Castrol, Berkshire, UK), were evaluated by our rotational diffusometer and a commercial rotational viscometer (Rheolab QC, Anton Paar, GRAZ, Austria). Each oil product was measured thrice. 

### 2.3. Fabrication of Janus Microbeads

**Figure 3:**
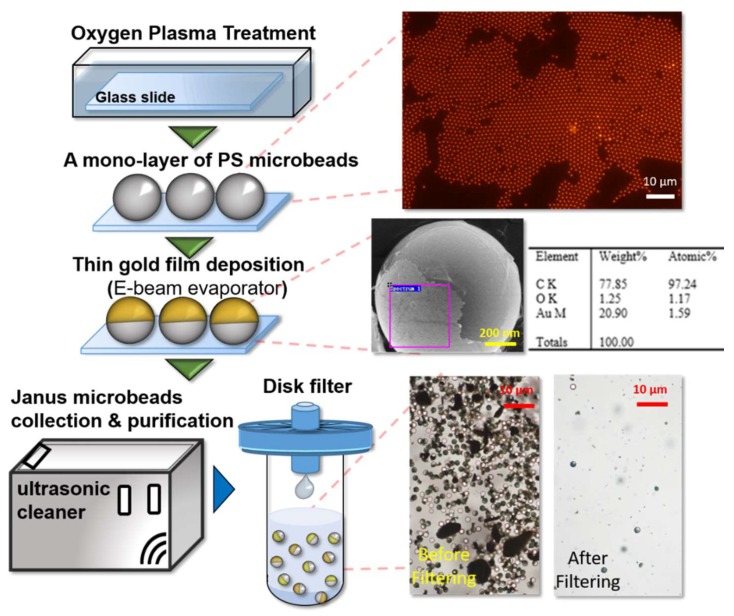
Fabrication procedure of the Janus microbeads. The flow chart is narrated in an order from the top to the bottom. A glass substrate is firstly treated with oxygen plasma to enhance hydrophilicity. Next, a drop of fluorescent PS suspension is dried on the glass slide to form a mono-layer of microbeads by self-assembly. A thin film of gold (30 nm) is deposited on the top of the mono-layer of microbeads by E-beam evaporation. The SEM image and X-ray spectrum confirm the gold coating on the microbead. At last, Janus microbeads are collected from the glass substrate by sonication and purified through a filtering process.

Considering that microbead size can be a factor altering rotational Brownian motion, size uniformity must be carefully controlled in a rotational diffusometer. To fabricate uniform Janus microbeads with a diameter as small as 1 μm, we adopted the procedure of metallic half-shells [41] (Figure 3). Fluorescent PS beads 1 μm in diameter were purchased from Thermo Fisher Scientific (Waltham, MA, USA, R0100, Excitation: 541 nm/Emission: 611 nm); the size variation of these beads was well defined below 5% coefficient of variation. First, a glass slide was treated with oxygen plasma to create a superhydrophilic surface to avoid the coffee-ring effect during evaporation. Second, a small drop of the microbead suspension was allowed to dry on the surface of the glass slide. Within 30 min, a well-spread mono-layer of microbeads formed on the slide. Next, a thin layer of 30 nm gold film was deposited on the mono-layer of microbeads via an e-beam evaporator. The Janus microbeads were subsequently harvested from the glass slide by sonication for 30 min and resuspended in water with 5% (*v*/*v*) Tween 80. The collected Janus microbead suspension was purified by passing through a filter disk with a 5-μm pore size. The concentration of the colloidal suspension significantly dropped after filtration. Finally, centrifugation was performed thrice to boost the concentration of the suspension up to 2 × 10^9^ beads/mL. As shown in the SEM image in Figure 3, nearly half of the surface of the PS microbeads was covered by the metallic film, as expected. Despite irregular edges of the coating, the non-uniformity influenced only the visibility of blinking signal. The time-elapsed correlation intensity would not be seriously altered since the blinking frequency was not shifted. In addition, the random variation was mitigated when more microbeads were put into calculation. As a result, averagely 40–70 microbeads were analyzed in each image to overcome the uncertainty for each data point. The elements on the microbeads were examined by X-ray spectrometry (EMAX HR, Horiba Scientific, Kyoto, Japan), and the small fraction of gold shown in the table of elements in Figure 3 confirmed successful coating. The gold film coated on each particle was quite firm and sturdy. No chemical interactions between the gold film and liquid media were observed. The shelf life of Janus microbeads was more than weeks according to our observation. In addition, the gold surface provides an ideal substrate for future biological modifications.

## 3. Results and Discussion

### 3.1. Evaluation of Rotational Brownian Motion with Different Microbead Sizes

**Figure 4:**
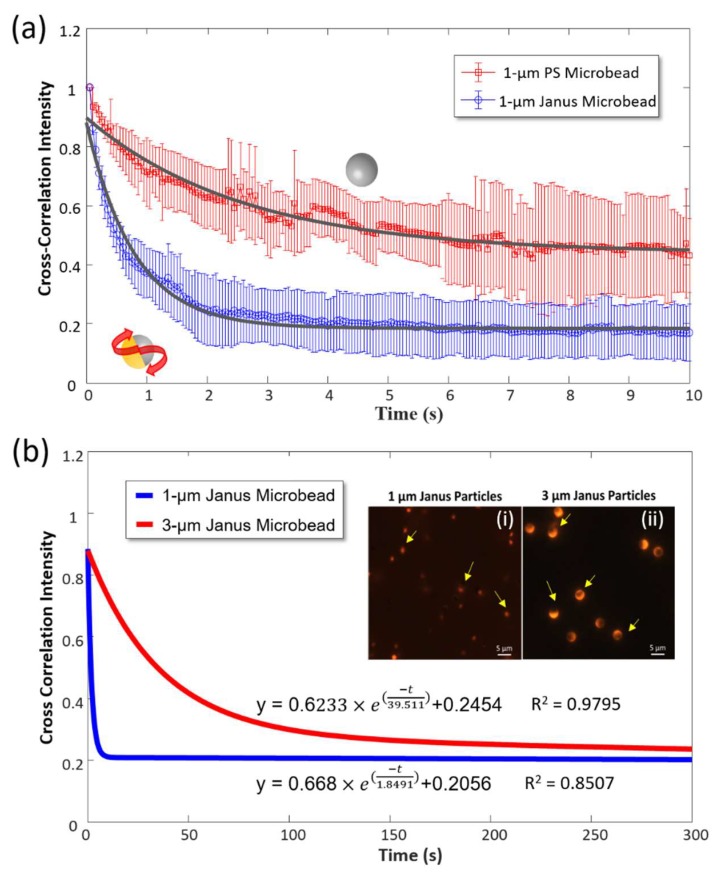
(**a**) Time-elapsed correlation intensity of 1-μm PS and Janus microbeads. (*n* = 3) (**b**) Time-elapsed correlation intensity of 1 and 3-μm Janus microbeads. (*n* = 3) The solid lines here represent logarithmic fitting curves. In the insets, some of the microbeads are indicated by yellow arrows.

First, the time-elapsed correlation intensities of pure 1-μm PS and Janus microbeads were compared (Figure 4a). All correlation intensity values were normalized by the initial one for a better comparison. The Janus microbeads significantly outperform the pure PS microbeads in terms of SNR of correlation intensity. The intensity change of the Janus microbeads is nearly double that of the PS microbeads, and the intensity variation of the former is smaller than that of the latter. The overall results indicate that rotational Brownian motion measured from the Janus microbeads is the dominant signal in the correlation intensity. Subsequently, two Janus microbead sizes, 1 and 3 μm, were evaluated with the rotational diffusometer. Normalized logarithmic fitting curves are depicted here to show time-elapsed correlation intensity of both Janus microbeads (Figure 4b). The insets (i) and (ii) in Figure 4b illustrate the microscopic fluorescent images of the beads. A crescent-shaped fluorescence can be explicitly observed in the 3-μm Janus microbeads, and the time-elapsed cross-correlation intensity of the 1-μm Janus microbeads shows a much steeper decline compared with that of the 3-μm Janus microbeads. The ratio of measured characteristic correlation times between the 3 and 1-μm microbeads is 21.37, which is close to the predicted ratio of 27 derived from Equation (4). While the decline in correlation intensity may be seriously compromised by a high viscosity or large bead size, the characteristic correlation time may still be found by extending the measurement duration. Considering that the 1-μm microbeads have a shorter correlation time in water, these beads were used for subsequent studies, unless otherwise mentioned.

### 3.2. Calibration of the Viscosity of Glycerol–Water Mixtures

**Figure 5:**
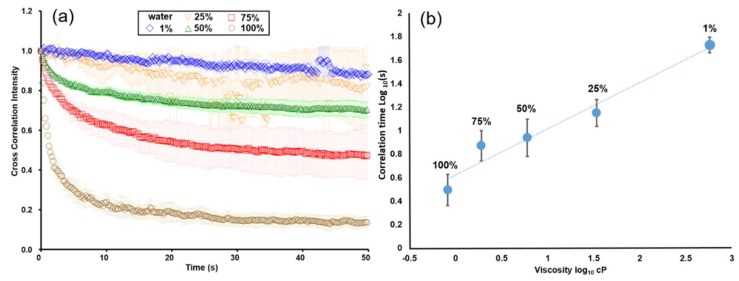
(**a**) Time-elapsed correlation intensity of glycerol–water mixtures of different ratios. The water contents of these mixtures ranged from 1% to 100% (*v*/*v*). The error bars are standard deviations obtained from data points measured from three different sites in the same medium. (*n* = 3) (**b**) Calibration curve of characteristic correlation time versus viscosity. The error bars represent standard deviations obtained from data points measured from three independent glycerol-water solutions. (*n* = 3).

Glycerol was mixed with deionized water to produce a series of mixtures ranging from 1% to 100% (*v*/*v*) water. The room temperature was maintained at 27 °C, but the real temperature inside the microchip was slightly higher than that of the environment due to light illumination. As a result, the corrected temperature was 28.5 °C. The standard viscosity of glycerol-water mixtures was derived from the literature [42,43]. The decline in time-elapsed correlation intensity significantly slows down when the water content is less than 75% (*v*/*v*) (Figure 5a). Because of the slow decline in a higher viscosity solution, an extended measurement up to 150 s was conducted especially for the mixture of 1% (*v*/*v*) water content to allow precise curve fitting. To avoid inconsonant display, however, the portion longer than 50 s was not shown in the figure. By deriving the characteristic correlation times from Figure 5a, a calibration curve showing the relationship between the correlation times and corresponding viscosities was plotted (Figure 5b). This curve was then used to convert the following measurements into final viscosities. 

### 3.3. Viscosity Measurements of Oil Products and their Degradation

**Figure 6:**
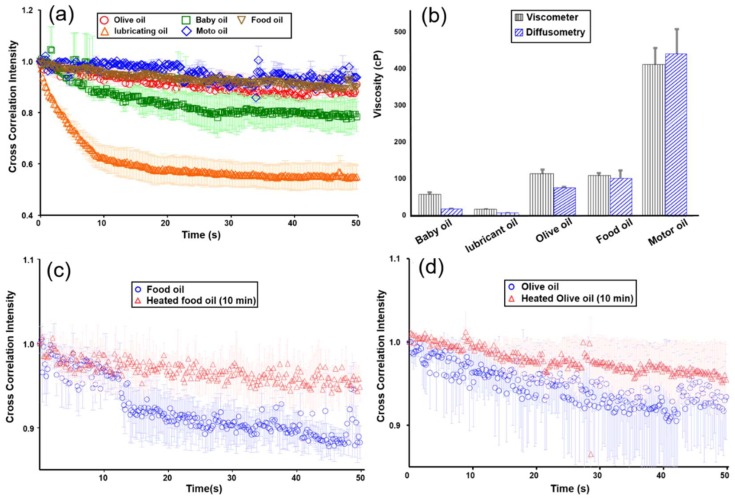
(**a**) Time-elapsed correlation intensity of five selected oil products. (*n* = 3); (**b**) Comparison of measured viscosities between the proposed rotational diffusometer and a commercial rotational viscometer. (*n* = 3); (**c**,**d**) Time-elapsed correlation intensities of food oil and olive oil before and after heat treatment. (*n* = 3).

The proposed technology was applied to five oil products, including lubricant oil, baby oil, food oil, olive oil, and motor oil, to demonstrate its applications. Here, 1-μm Janus microbeads were mixed with all of the oil products before measurement. Surfactant (5% (*v*/*v*); Tween 80, Merck, Darmstadt, Germany) was added to a total 10 μL of the colloidal suspension. 30-min sonication was then implemented to ensure thorough dispersion. The change in viscosity of the oils before and after addition of the trace surfactant was negligible. The temperature of the microchip was maintained at 28.5 °C. Each oil sample was measured thrice, and only 2 μL was needed for each measurement. The measurement duration was increased to 50 s in response to the significantly increased viscosity of all oil products (Figure 6a). A rotational viscometer was used to provide reference data for comparison. As predicted, the viscosities (lubricant oil: 1.7 cP, baby oil: 16.9 cP, olive oil: 74.0 cP, food oil: 97.5 cP, and motor oil: 434.5 cP) derived from the measured characteristic correlation times show comparable data with those (15.7, 56.0, 104.6, 112.2, and 409.2 cP, respectively) measured by the rotational viscometer (Figure 6b). The differences may be resulted from the calibration curve and gaps between local and bulk viscosities. The accuracy of calibration curve can be improved by increasing the number of measurement. Notably, surface tension only matters when Janus microbeads need to be resuspended in the oil media. As long as microbeads are well mixed with oil, translational and rotational Brownian motion is mainly dominated by viscosity according to both Stoke-Einstein and Stokes-Einstein-Debye relations. This reasoning was also supported by the good agreement between our diffusometer and the rotational viscometer as shown in Figure 6b.

Another evaluation was conducted to verify oil viscosity changes after heating at a high temperature. Prior research [3] states that fatty acids in oil degrade after deep frying due to oxidation and polymerization. An increase in saturated fatty acid content, therefore, influences the physical properties of oil, such as its viscosity. The oil viscosity shift can serve as an indicator of the degradation of oil quality after heat treatment. Food oil and olive oil were selected for the degradation experiment. Both oils were heated in a frying pot on a hotplate at 1000 W for 10 min to a temperature above 100 °C. Smoke was observed. Both food oil and olive oil exhibit increases in viscosity from 35.4 and 59.8 cP to over 1325.2 and 123.6 cP, respectively (Figure 6c,d). Olive oil tends to undergo less extensive viscosity changes than food oil, thereby implying good stability during the high-temperature treatment. This finding is consistent with those in previous studies [3,4].

## 4. Conclusions

Micro-volume viscosity measurements have long been a challenging task in many fields because existing viscometers cannot provide an appropriate solution. This study presents a rotational diffusometer using Janus microbeads to estimate oil viscosity. The Janus microbeads were fabricated by depositing a thin gold film on the half-surface of fluorescent PS microbeads and produced blinking signals in response to different media according to the Stokes–Einstein–Debye relation. A plot of time-elapsed correlation intensity was fitted to an exponential curve to enable calculation of the characteristic correlation time of the measured data. Compared with translational Brownian motion, rotational Brownian motion was proven to enhance the SNR of correlation intensity. A calibration curve covering the viscosity range from 0.8 cP to 574.8 cP was then obtained from glycerol–water mixtures. Five common oil products, including lubricant oil, baby oil, food oil, olive oil, and motor oil, were investigated, and their viscosities were determined by our diffusometer to be 1.7, 16.9, 74.0, 97.5, and 434.5 cP, respectively; these values are comparable with those measured by a commercial rotational viscometer (15.7, 56.0, 104.6, 112.2, and 409.2 cP, respectively). When food oil and olive oil were heated at a high temperature above 100 °C for 10 min, their viscosities increased from 35.4 and 59.8 cP to 1325.2 and 123.6 cP, respectively. These viscosity increases imply an increase in saturated fatty acid composition, resulting in degradation of oil quality. Olive oil appeared to be more invulnerable to high temperatures than food oil due to its lower viscosity shift. Evaluations suggest that the proposed rotational diffusometer can be a promising tool for micro-volume viscosity monitoring of Newtonian fluids.
